# Prioritizing Candidate Disease Metabolites Based on Global Functional Relationships between Metabolites in the Context of Metabolic Pathways

**DOI:** 10.1371/journal.pone.0104934

**Published:** 2014-08-25

**Authors:** Desi Shang, Chunquan Li, Qianlan Yao, Haixiu Yang, Yanjun Xu, Junwei Han, Jing Li, Fei Su, Yunpeng Zhang, Chunlong Zhang, Dongguo Li, Xia Li

**Affiliations:** 1 College of Bioinformatics Science and Technology, Harbin Medical University, Harbin, P. R. China; 2 School of Medical Informatics, Daqing Campus, Harbin Medical University, Daqing, P. R. China; 3 School of Biomedical Engineering, Capital Medical University, No. 10 You An Men Wai Xi Tou Tiao, Beijing, P.R. China; Harbin Medical University, China

## Abstract

Identification of key metabolites for complex diseases is a challenging task in today's medicine and biology. A special disease is usually caused by the alteration of a series of functional related metabolites having a global influence on the metabolic network. Moreover, the metabolites in the same metabolic pathway are often associated with the same or similar disease. Based on these functional relationships between metabolites in the context of metabolic pathways, we here presented a pathway-based random walk method called PROFANCY for prioritization of candidate disease metabolites. Our strategy not only takes advantage of the global functional relationships between metabolites but also sufficiently exploits the functionally modular nature of metabolic networks. Our approach proved successful in prioritizing known metabolites for 71 diseases with an AUC value of 0.895. We also assessed the performance of PROFANCY on 16 disease classes and found that 4 classes achieved an AUC value over 0.95. To investigate the robustness of the PROFANCY, we repeated all the analyses in two metabolic networks and obtained similar results. Then we applied our approach to Alzheimer's disease (AD) and found that a top ranked candidate was potentially related to AD but had not been reported previously. Furthermore, our method was applicable to prioritize the metabolites from metabolomic profiles of prostate cancer. The PROFANCY could identify prostate cancer related-metabolites that are supported by literatures but not considered to be significantly differential by traditional differential analysis. We also developed a freely accessible web-based and R-based tool at http://bioinfo.hrbmu.edu.cn/PROFANCY.

## Introduction

A major challenge in today's medicine and biology is to identify the key metabolites associated with complex diseases. Because metabolites are modulated by genetic and environmental perturbations; their alterations in the concentration can reflect disturbed metabolic functions and reveal novel physiological and pathophysiological information, which can not be obtained directly from the genomics, transcriptomics, and proteomics [1]–[3]. Metabolomics, which is a quantitative description of all endogenous metabolites found in cells and body fluid, aims at characterization of the metabolome under different conditions [for example, diseases] [2], [4], [5]. Metabolomics can not only help us illustrate the underlying molecular disease-causing mechanisms but also gain broad recognition in discovery of metabolic signatures [biomarkers] for disease diagnosis [1], [4], [5].

The main technology of metabolomics is using nuclear magnetic resonance (NMR) spectroscopy or liquid/gas chromatography-mass spectrometry (LC/GC-MS) to profile and quantify concentrations of hundreds of metabolites simultaneously [1], [2], [4], [6]. The metabolic profiles have been widely applied in disease related metabolites identification and diagnostic biomarker discovery [1]. However, these high-throughput techniques have several limitations. For example, it is difficult to determine quantitative information from peak integration due to the different ionization ability of various metabolites and the sensitivity of these techniques is not satisfactory, which can lead to false positive metabolomics results [1], [7]. Therefore, it is necessary to develop a computational method to prioritize the candidate disease metabolites from metabolomics profiles.

The development and completeness of some high quality metabolic network databases have led to availability of computational method for prioritization of metabolites. The metabolites rarely function in isolation; rather, they carry out biological functions together through thousands of biochemical reactions which organize into intricate metabolic network [8], [9]. Thus, metabolites in the consecutive reactions are functionally interrelated [8]. As a consequence, the impact of a disease on human metabolism is not always restricted to one or two reactions but is potentially spread among the functionally related metabolites in the metabolic network [8], [10], [11]. Therefore, adjacent functional related metabolites tend to relate to the same or similar disease [8]. Meanwhile, metabolites in the network are not equally functionally related. Some strongly related metabolites in the same functional module, for example a metabolic pathway, together exert a special biological function [12]. The abnormity of metabolites in one module [pathway] tend to inactivate a special biochemical function, thus leading to the same or similar disease [12]–[15].

**Figure 1:**
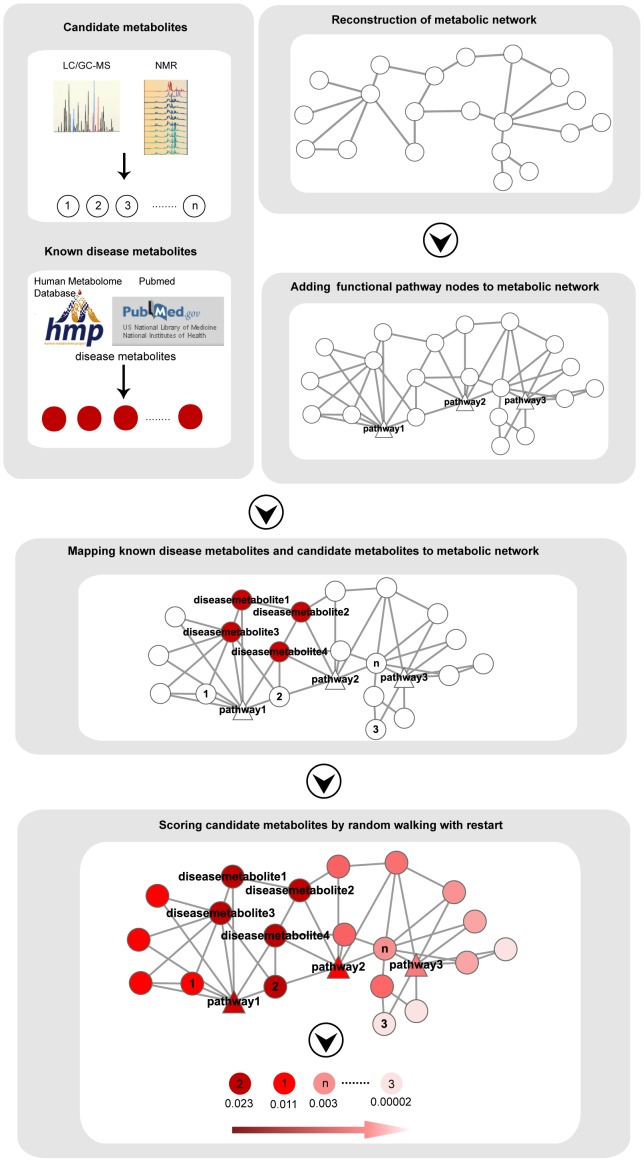
Schematic of the PROFANCY. We firstly reconstruct metabolic networks based on the structure data from KEGG or EHMN database and add functional pathway nodes in this metabolic network. We then map the known disease metabolites (seed nodes) and candidate metabolites into the above network. After that, we extend random walk with restart (RWR) method to this network. Finally, we can rank the candidate metabolites according to the steady probability of RWR.

With these understandings, we developed a global computational method called PROFANCY (PRioritization of candidate metabOlites using Functional relAtioNships in the Context of metabolic pathwaY) to prioritize candidate disease-related metabolites based on the assumption that metabolites associated to the same disease are functionally related in the context of metabolic pathways (see Materials and methods and Figure 1). In our method, we firstly reconstructed a global metabolic network in which nodes presented metabolites and two metabolites were connected if they were belonging to the same reaction according to the pathway structure data from the KEGG or EHMN database [16]–[18]. Considering the fact that the metabolites related to the same disease tend to be functional modularized [in one pathway] in metabolic network [12], we took advantage of the functional modularity of metabolic network according to different pathways. Thus we added functional pathway nodes (FPN) on the above metabolic network and connected these nodes to all the metabolites belonging to the corresponding pathway. Finally, we employed the random walking with restart [RWR] method on this “functional module-enhanced” network, using the known disease related metabolites as seed nodes from the Human Metabolome Database (HMDB) (Figure 1) [19], [20]. We applied the PROFANCY to 71 diseases and achieved an AUC value up to 0.895. We also applied this method on different disease classes and achieve an AUC value over 0.95 in 4 classes. To investigate the robustness of the PROFANCY, we repeated all the analyses in another metabolic network reconstructed according to the EHMN database and obtained the stable results [16]–[18]. Then we assessed the importance of functional pathway nodes and found that these nodes contributed to the good performance and robustness of PROFANCY. In the following case studies, we applied our method to (i) prioritize candidate metabolites for Alzheimer's disease; (ii) prioritize the metabolites from metabolomic profiles of prostate cancer. We identified a potential prostate cancer related metabolite which supported by literatures but not considered to be significantly differential in metabolomic profiles. We also developed a freely accessible web-based and R-based tool at http://bioinfo.hrbmu.edu.cn/PROFANCY.

## Materials and Methods

### Known disease metabolites

The known disease–metabolite associations were extracted from the Human Metabolome Database (HMDB) which collected detailed information of small molecule metabolites found in the human body, including their related disease phenotype information described in entries in OMIM [19]. We removed the diseases which have less than two related metabolites. We only retained the metabolites which existed in the reconstructed KEGG and EHMN metabolic networks (see below). All HMDB metabolite identifiers were converted to identifiers in KEGG. Finally, we obtained 71 diseases and 338 disease related metabolites (HMDB vision 2.5). These metabolites were considered as known disease related metabolites (seed nodes).

### Reconstruction of metabolic network

For prioritizing disease metabolites in a global view, we reconstructed an undirected metabolic network in which nodes represented metabolites and two metabolites were connected if they were in the same reaction. To do this, we extracted the pathway structure information from two databases: KEGG [18] and the EHMN [16], [17], and reconstructed two networks to get robust results. For KEGG database, we downloaded the manually collected reaction information from published materials. To obtain specific relations between metabolites, we deleted some common metabolites such as H2O, CO2, and so on (see Table S6) [14]. Finally, we got 3617 nodes and 4771 edges in KEGG metabolic network. The EHMN database is a high-quality human metabolic network manually reconstructed by integrating genome annotation information from different databases and metabolic reaction information from literature [16], [17]. We downloaded the SBML files from EHMN website and extracted the metabolic reactions. After the same dealing steps as KEGG metabolic network, we got 1629 nodes and 5239 edges in the EHMN metabolic network.

### PROFANCY

The PROFANCY could prioritize candidate disease metabolites by fully exploiting the global functional similarity of metabolites and the functionally modules of metabolic network. To take advantage of global functional similarity between metabolites, we employed RWR method, introduced by Kohler, S et. al [20], which was defined as an iterative random walker's transition from its current node to its neighbours starting at a given source node 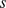, with a additionally allowable restart of the walk in each step at the node 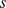 with probability 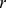 (In this study, we set 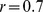 and this parameter would be discussed in the following sections). Formally, the random walk with restart is defined as:
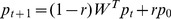
(1)In this formula, 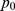 is the initial probability vector in which each seed node has equal probabilities, and the 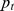 is a vector in which the 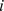
th element describes the probability of being at node 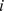 at time step 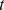. 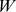 is the transition matrix and 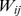 is the transition probability from node 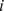 to node 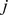 which would be described later. The candidate metabolites rank was obtained when the difference between 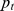 and 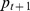 fell below 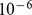.

Some studies indicated that metabolites in the same modules (pathways), together exerting a special biological function, were prone to lead to a special or similar disease [8], [12]. To exploit functional modularity of metabolic network, we added functional pathway nodes to above two networks. Firstly, we downloaded metabolites–pathway associations from the KEGG database [18]. Then we searched the metabolites belonging to the same pathways in metabolic networks. Finally, we added the functional pathway nodes in both metabolic networks and made these nodes connect to the metabolites which belonging to the corresponding pathway (Figure 1). There were 145 or 133 functional pathway nodes in KEGG or EHMN metabolic network, respectively. In this functional module-enhanced metabolic network, there were two kinds of links including the links between metabolites nodes and links between functional pathways nodes and metabolites nodes. Suppose 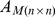, 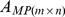, 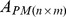 and 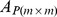 are adjacency matrix for metabolite links, the metabolite-pathway links, pathway-metabolite links and pathway-pathway links, respectively, where 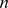 and 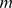 represent the number of metabolite and functional pathway nodes. There were no links edges between functional pathway nodes, so here 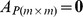. The adjacency matrix of the module-enhanced metabolic network can be represented as 
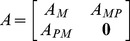
. Then let 
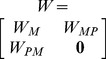
 be the transition matrix of the functional module-enhanced metabolic network, where 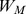 is transition matrix of metabolites and 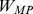, 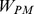
are metabolite-pathway transition matrix and pathway-metabolite transition matrix, respectively. The transition probability from a metabolite node 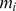 to a pathway node 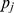 can be described as
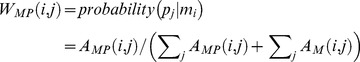
(2)Similarly, the transition probability from 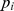 to 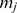 can be described as
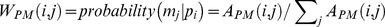
(3)The probability of the random walker transition from a metabolite node 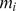 to another metabolite node 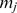 can be defined as
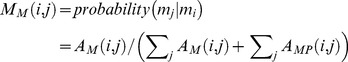
(4)The initial probability vector is represented as 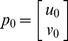. Let 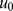 and 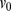 represent the initial probability of metabolite nodes and pathway nodes, respectively. The 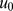 is constructed such that equal probabilities are assigned to the seed metabolite nodes in the metabolic network, with the sum of the probabilities equal to 1. Here, the initial probability of pathway nodes (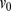) is equal to 0. This is equivalent to letting the random walker begin from each of the known disease metabolites with equal probability. Here, the initial probability of pathway nodes is equal to 0. We put the transition matrix 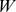 and initial probability 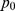 into the iterative equation (1) and after above steps, the steady probability 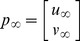 is obtained, in which 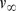 and 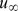 is the steady probability of functional pathway nodes and metabolite nodes. The steady probability is obtained at query time by performing the iteration until the difference between 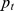 and 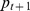 fell below 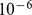. Then candidate metabolites can be ranked based on the steady probability 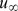 (Figure 1).

### Performance measurement

To access the performance of PROFANCY, we used leave-one-out cross-validation method on every disease metabolite. For each disease, each of the known metabolites was taken as one test case. For each test case, the remaining known disease metabolites were used as seed nodes. The held out metabolite and other metabolites in the network were considered as candidates. After the implementation of RWR method, each metabolite in the network was assigned a probability value. Then we could rank test metabolite with the other nodes in the network together. Therefore, for each test metabolite of every disease, we could obtain a rank list. Taking all rank lists of all disease metabolites together, we could calculate the ratio of the known disease metabolites which ranked in top n%.

The receiver operator characteristic (ROC) curve could also be plotted and the area under this curve (AUC) could be calculated according to above results. The ROC curve plots the true-positive rate (TPR) versus the false-positive rate (FPR). For evaluating rankings of disease-metabolite predictions, here ROC curves could be interpreted as a plot of the frequency of the disease metabolites above the threshold versus the frequency of disease metabolites below the threshold, where the threshold is a specific position in the ranking [20].

## Results

In this section, we first assessed the performance of the PROFANCY method on 71 diseases which could be grouped into 16 classes. Then we assessed the robustness of the PROFANCY. After that, we investigated the contribution of functional pathway nodes in the prioritization process of PROFANCY. In the following two case studies, we predicted novel potential disease metabolites for Alzheimer's disease using PROFANCY. Furthermore, we applied our method in prioritizing the metabolites from metabolomic profiles of prostate cancer.

### Performance of PROFANCY

**Figure 2:**
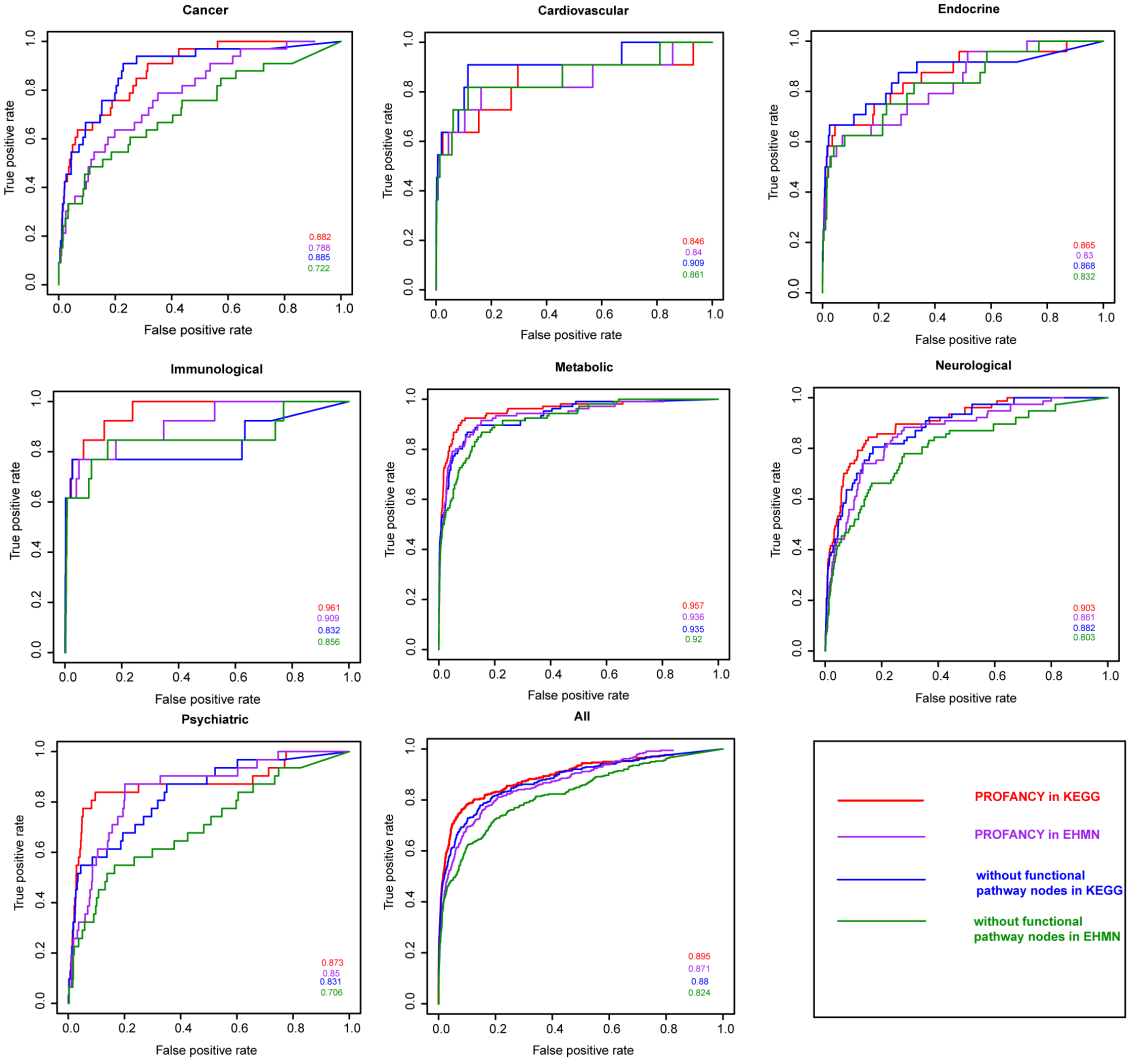
The ROC curve of 7 disease classes and all diseases with or without functional pathway nodes in two metabolic networks.

To assess the performance of our method, we performed a validation with 338 known disease metabolites associated with 71 diseases obtained from the HMDB database (see Materials and Methods) [21]. For 71 diseases, the AUC value was up to 0.895 (Figure 2). Additionally, 95% known disease related metabolites were ranked in top 50%; and over 80% (267) know disease related metabolites were ranked in top 10% (Table S1). Even in top 5%, there were still 64% known disease related metabolites in the KEGG metabolic network (Figure S1).

We found that our method have outstanding performance on some diseases. For example, all the known metabolites of maple syrup urine disease, lesch-nyhan syndrome and propionic academia were ranked in top 10%, respectively (Table S1). Majority (37 in 44) of the known metabolites of Alzheimer's disease was ranked in top 10%; 19 of 22 known metabolites for schizophrenia were also ranked in top 10% (Table S1). These diseases with better performance were belonging to metabolic class or closely related to metabolism. Then we questioned that whether the metabolic diseases could achieve the best performance and how the PROFANCY performed on other disease classes. To further investigate this, we grouped the 71 diseases into 16 classes and calculated their AUC values. Of all 16 disease classes, the PROFANCY achieved an AUC value over 0.7 in 12 classes in which 4 classes could achieve over 0.95 (Table 1). The metabolic diseases have the fourth highest AUC value of 0.957. We found that top 3 disease classes (respiratory, muscular and immunological classes with an AUC value of 0.999, 0.973 and 0.961, respectively) were closely related to abnormal metabolism. For example, cystic fibrosis, which was belonging to respiratory class, was found to have abnormalities in lipid, oxidants, bile acid, and amino acid metabolic processing [22]; Addison's Disease (an immunological diseases) are caused by the dysfunction in biosynthesis of glucocorticoids and mineralocorticoids [23]. This outstanding performance of PROFANCY on metabolic and metabolism-related diseases might be due to the closer association between the pathogenesis of these diseases and dysfunction of corresponding metabolic pathways [8], [13]. We also noticed that AUC value was relatively lower for some disease classes. For example, the AUC of developmental diseases and hematological diseases were lower than 0.6. We found that there was only one disease in the above two classes respectively and each disease only had two known related metabolites. The incomplete metabolites data might limit the performance of our method on the two disease classes.

**Table 1:** The AUC value of 16 disease classes.

Disease Class	AUC value			
	KEGG without FPN	PROFANCY in KEGG	EHMN without FPN	PROFANCY in EHMN
Metabolic	0.935	0.957	0.92	0.936
Neurological	0.882	0.903	0.803	0.861
Cardiovascular	0.909	0.846	0.861	0.84
Endocrine	0.868	0.865	0.832	0.83
Immunological	0.832	0.961	0.856	0.909
Muscular	0.988	0.973	0.293	0.797
Psychiatric	0.831	0.873	0.706	0.85
Cancer	0.885	0.882	0.722	0.788
Connective tissue	0.859	0.838	0.794	0.793
Developmental	0.488	0.578	0.466	0.556
Gastrointestinal	0.788	0.821	0.774	0.948
Multiple	0.7	0.632	0.712	0.776
Respiratory	0.999	0.999	0.974	0.991
Renal	0.934	0.936	0.879	0.881
Nutritional	0.681	0.639	0.721	0.684
Hematological	0.343	0.425	0.809	0.612
All	0.88	0.895	0.824	0.871

FPN = functional pathway nodes.

### Investigating the robustness of PROFANCY

Another important issue of our method lies in the robustness of the PROFANCY. We investigated the robustness of our method from following aspects: (i) repeating all analyses in another metabolic network reconstructed from the EHMN database; (ii) randomizing the metabolic networks; (iii) perturbation of the candidate metabolites; (iv) setting different value of the restart probability 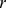.

At first, we reconstructed another metabolic network from the EHMN database (see Materials and Methods) and repeated the above analyses. We found that the results of PROFANCY were stable in the EHMN metabolic network. For 71 diseases, the AUC value was 0.871. There were 95%, 80% (230) and 47% known disease related metabolites ranked in top 50%, 10% and 5%, respectively, in the EHMN metabolic network (Figure S1 and Table S2). Of 16 disease classes, the AUC values of 13 classes were more than 0.7 and 4 classes could achieve up to 0.9 (Table 1). Among them, highest AUC value (0.991) was also obtained from the respiratory diseases and the AUC value of metabolic diseases (0.936) was close to that in the KEGG metabolic network (Table 1).

We next assessed the robustness of PROFANCY after perturbation of metabolic network. After removing edges in the original metabolic network from a percent of 10% to 90%, we calculated the AUC value in these incomplete networks, respectively. We found that the PROFANCY had strong resistance against the incompleteness of network–the AUC value only had a slight decline (about 0.005 or 0.003) when deleting 10% edges of the KEGG or EHMN network (Table S3); Even when we deleted 70% edges, our method could keep a relatively high AUC value about 0.8 in both networks.

We also investigated that whether our method still have stable performance under the perturbation of candidate metabolites. For each disease, we randomly selected 99 metabolites as candidates from original candidate metabolites (see “Performance measurement” in Materials and Methods). We obtained similar AUC values of all 16 disease classes in both networks (Table S4). Finally, to investigate the influence of 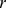 value, we set it at 0.1, 0.3, 0.5, 0.7 and 0.9, and then calculated the AUC value in KEGG and EHMN metabolic networks, respectively. For each 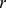 value setting, PROFANCY method had robust performance (Table S5). In this work, we set it to 0.7.

### Contribution of functional pathway nodes in the prioritization

In the PROFANCY, we added functional pathway nodes (FPN) to sufficiently exploit functional modularity of metabolic network and thus to enhance prioritizing ability. To assess the contribution of FPN in the process of prioritization, we compared the performance of PROFANCY with FPN to that without these nodes. We deleted these nodes and then prioritized the candidate metabolites on the original metabolic network only. After we performed the validation using the same data as we did above, we found that in both metabolic networks, there were more known disease metabolites which ranked in top 5%, 10% and 50% by PROFANCY than that deleting the FPN (Table S2). For example, in the KEGG metabolic network, 267 known disease metabolites were ranked in top 10% by PROFANCY. However, when deleting the FPN, there were only 245 metabolites ranked in top 10%.

Furthermore, the AUC value of PROFANCY for 71 diseases was higher than that without FPN in both networks (Table 1). We further compared the AUC values in 16 disease classes. In majority disease classes, the AUC values had improved with FPN compared to that without these nodes in the EHMN network (Table 1). Among them, the AUC value of some classes had improved to a relatively great extent. For example, the AUC value of immunological diseases had an improvement more than 0.1 (rising from 0.832 to 0.961); the psychiatric diseases also have a great improvement about 0.15 in the EHMN network. Surprisingly, the AUC value of metabolic diseases had a little (about 0.02) improvement. The reason might be that the metabolites associated to metabolic diseases had relatively closer functional relationships and always concentrated in a local region (continuous reactions) of metabolic pathway. Although we added the FPN, the AUC value of these diseases might not have a great improvement due to the already existing closely functional modularity. However, unlike typical metabolic diseases, the metabolites associated to other diseases might distribute in a relatively larger scale throughout the metabolic network and have a relatively loose connectivity. For example, malaria had three known metabolites: kynurenate, quinolinate and pipecolic acid which were belonging to different pathways. Kynurenate and quinolinate participated in the tryptophan metabolism (path: 00380), and quinolinate and pipecolic acid participated in the biosynthesis of alkaloids derived from ornithine, lysine and nicotinic acid (path: 01064). In this condition, the performance was not good without the FPN due to relatively loose connectivity between the above metabolites (none of three metabolites were ranked in top 10%, see Table S1). On the contrary, in the PROFANCY we added two functional pathway nodes to enhance the connectivity between the 3 disease metabolites metablites which belonged to the same or different pathways. In this condition, the performance would be improved due to the enhanced connectivity between disease metabolites–all three metabolites were successfully ranked in top 10% (Table S1).

The FPN contributed not only to the above improvement but also to the robustness of PROFANCY. We found that the FPN might contribute to the strong resistance against incompleteness of network. The AUC value in had a larger decline in the incomplete networks without FPN than that with FPN. For example, when deleting 10% edges of the KEGG network with FPN, the AUC had only a slight decline of 0.005; but in the network without FPN, the decline would have a six-fold (0.036) amplification (Table S3). This indicated that the FPN could maintain a part of functional relationships between disease metabolites even though we removed parts of edges in the metabolic networks.

### Case study 1: predict potentially novel metabolites for Alzheimer's disease

**Figure 3:**
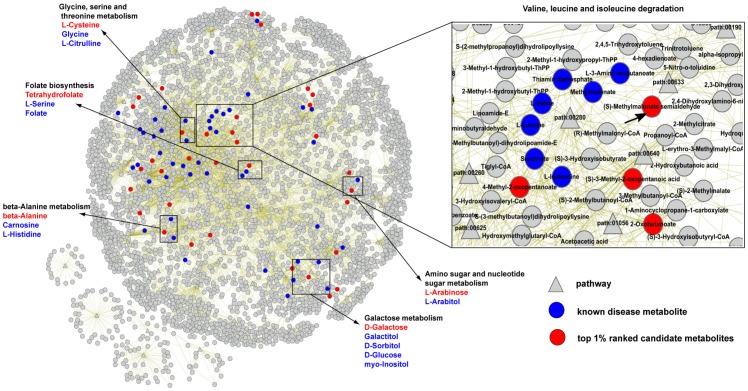
Top ranked candidate metabolites of Alzheimer's disease in the KEGG metabolic network. The top ranked candidate metabolites of AD are showed in the KEGG metabolic network. The gray, blue and red nodes represent candidate metabolites, known metabolites (seed nodes) and top 1% ranked candidates, respectively. The black boxes represent 6 candidates which ranked in top 10 and their connected functional pathway nodes in both metabolic networks. The right large box shows the pathway of “Valine, leucine and isoleucine degradation” which includes the metabolite (S)-Methylmalonate semialdehyde (arrow pointed).

Here we used the PROFANCY method to predict novel metabolic biomarkers for Alzheimer's disease (AD), which is considered to strongly associate with changes in systemic metabolite [24]–[26]. The known AD related metabolites from HMDB database were considered as seed nodes and other metabolites in the metabolic network were considered as candidates. After the implementation of PROFANCY on two metabolic networks, we found that 6 metabolites ranked in top 10 in both metabolic networks. These top ranked candidates and known AD related metabolites (seed nodes) tended to be in the same pathway (black boxes in Figure 3) and they might have closely functional relationships. After investigating the relations between top ranked candidates and AD from literatures, we found that 5 of 6 predictive metabolites were reported to highly relate to Alzheimer's disease or considered to be potential biomarkers for Alzheimer's disease [27]–[34] (Table 2). For example, some researches had reported that injection of D-galactose contributed to progression of AD in rat model [28], [29]. Furthermore, D-galactose and Glucose, which was a known AD related metabolite, participated in the same reaction in pathway of Galactose metabolism (pathway: 00052) (Figure 3).

**Table 2:** Top ranked Alzheimer's disease related metabolites by PROFANCY.

KEGG ID	Metabolites name	KEGG rank	EHMN rank	Reference
C06002	(S)-Methylmalonate semialdehyde	1	5	
C00097	L-cysteine	2	3	[27]
C00124	D-galactose	3	2	[28], [29]
C00099	Beta-alanine	4	10	[31], [34]
C00101	Tetrahydrofolate	7	9	[32]
C00259	L-arabinose	10	6	[33] [30]

The PROFANCY ranked (S)-Methylmalonate semialdehyde in the first place in the KEGG network. Surprisingly, to our knowledge, there were no literatures which directly explored the role of (S)-Methylmalonate semialdehyde (arrow pointed in right big box of Figure 3) in AD. However, we found that (S)-Methylmalonate semialdehyde participated to the process of “Valine, leucine and isoleucine degradation” (pathway: 00280; right big box of Figure 3). There were up to known 7 AD-related metabolites (blue nodes in right box of Figure 3) in this pathway, suggesting that this pathway might play an important role in AD. Furthermore, in this metabolic pathway, (S)-Methylmalonate semialdehyde could be reversibly converted to L-3-Amino-isobutanoate and Methylmalonate (which were both known AD-related metabolites) by 4-aminobutyrate aminotransferase (ABAT) and aldehyde dehydrogenases (ALDH), respectively. Studies indicated that the activity of ALDH was significantly increased in the patients suffering from AD and it might act as antioxidant enzymes in the oxidative stress which contributed to AD [35]. Also, It was reported that the activity of ABAT was correlated to certain neuropsychiatric disorders such as epilepsy and Alzheimer's disease [36]. These indicated that the concentration of (S)-Methylmalonate semialdehyde might fluctuate due to the cascading effect of above two enzymes and the concentration change of L-3-Amino-isobutanoate and Methylmalonate under the AD state. The above results suggested our method could only effectively capture known disease metabolites but also predict non-reported novel disease related metabolites.

### Case study 2: prioritize the candidate metabolites from metabolomic profile of prostate cancer

In this case, we applied our method to prioritize candidate metabolites from the metabolomics profiles of prostate cancer. To do this, we downloaded the GC/LC-MS profile of prostate cancer which contained hundreds of named metabolites across 42 tissues related to prostate cancer (16 benign adjacent prostates; 12 clinically localized prostate cancers and 14 metastatic prostate cancers) [37]. Then we mapped all the profiled metabolites to KEGG and EHMN metabolic networks. There were 109 metabolites which successfully mapped to above two networks. The seed nodes were known prostate cancer related metabolites from HMDB database. Of the above 109 metabolites, there were 4 metabolites were recorded as known prostate cancer related metabolites in HMDB database. The remaining 105 profiled metabolites were considered as candidates. After prioritization by PROFANCY, we generated a rank list of 105 candidate metabolites. We found that 6 candidates were ranked in top 10 in both networks (Table 3). These top ranked metabolites were all reported to associate with initialization and development of prostate cancer [38]–[54]. For example, PROFANCY ranked the sorbitol at the first place. Sorbitol was catalyzed by sorbitol dehydrogenase (SORD) whose expression was regulated by androgens, which were essential for the development of prostate cancer [51], [52]. The second ranked candidate was myo-inositol which had been considered as potentially important markers of prostate cancer in human EPS [40], [41], [47], [49].

**Table 3:** Top ranked prostate cancer candidate metabolites by PROFANCY.

KEGG ID	name	KEGG rank	EHMN rank	P-value (N vs T)	P-value (N vs M)	Reference
C00794	Sorbitol	1	3	0.731925	0.001756	[51], [52]
C00137	myo-Inositol	2	1	0.037338	1.65E-07	[40], [41], [47], [49], [50], [55], [56]
C00031	Glucose	3	6	0.02918	0.013403	[38], [48]
C00049	Aspartate	4	2	0.066092	0.015197	[45], [53]
C00095	Fructose	7	7	0.059253	0.003321	[39]
C00064	Glutamine	8	8	0.396531	0.333774	[42]–[44], [46], [54]

N vs T: normal samples vs localized cancer samples; N vs M: normal samples vs metastatic cancer samples.

**Figure 4:**
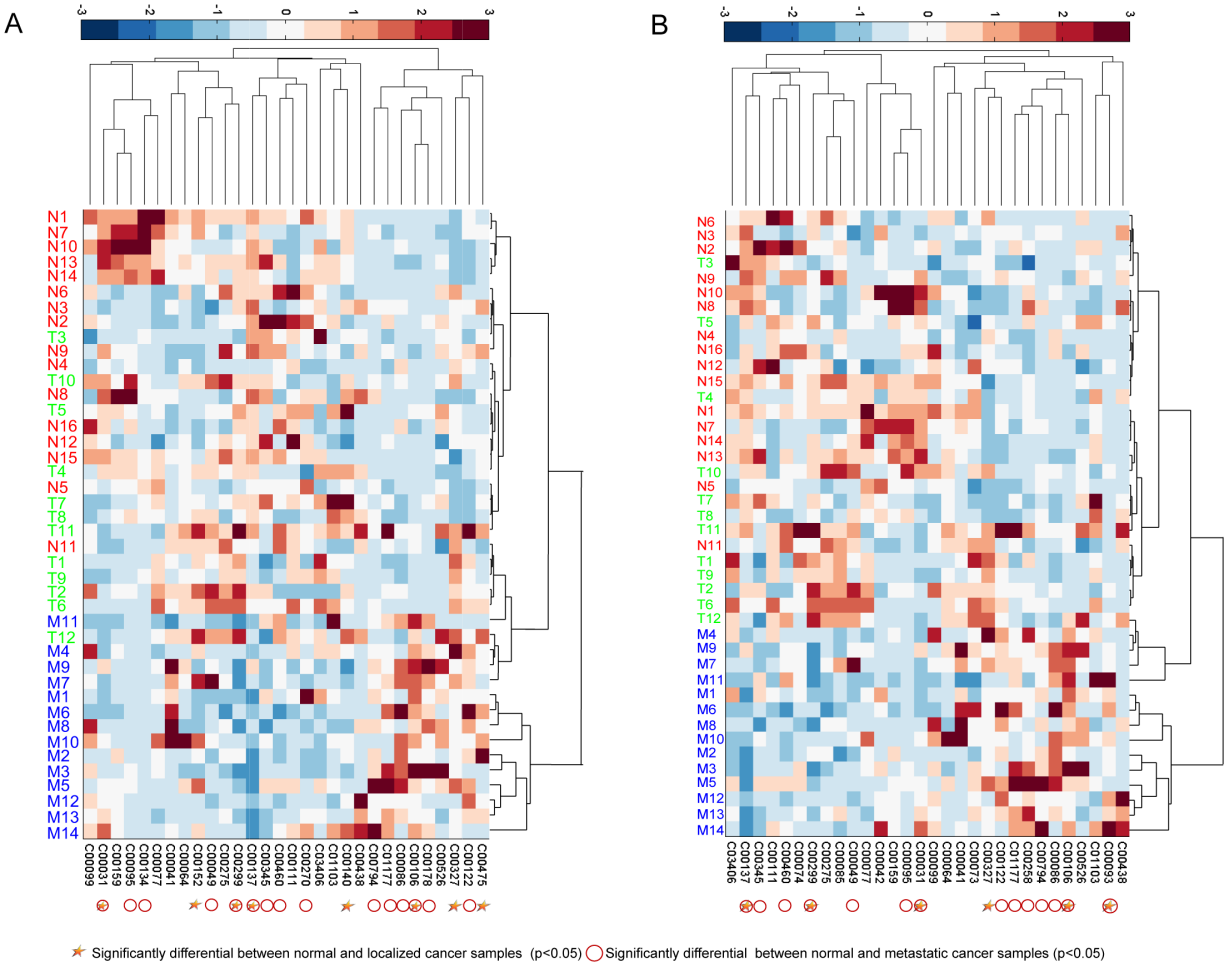
Cluster analyses of top 30 ranked prostate cancer candidate metabolites of from PROFANCY. Unsupervised hierarchical clustering of top 30 ranked candidate metabolites (columns) and samples (rows) is performed, and a heat map was generated. “N”, “T” and “M” represented the benign, clinically localized prostate cancer, or metastatic cancer samples, respectively. The top 30 ranked metabolites are from (A) KEGG metabolic network and (B) EHMN metabolic network.

We further investigated whether the top ranked metabolites generated by PROFANCY could be detected by the traditional differential analyses. To do this, we calculated the differential values of top 30 ranked metabolites by Wilcoxon rank-sum test between normal samples (benign adjacent prostates) and two kinds of cancer samples (localized cancer and metastatic cancer). We found that 5 metabolites were significantly differential in above 6 highly suspicious candidates (Table 3 and Figure 4) and over half of metabolites in top 30 ranked metabolites were significantly differential between normal and localized cancer samples or metastatic cancer samples (P-value<0.05; Figure 4), suggesting that PROFANCY could identify majority of significantly differential metabolites.

However, we also noticed that some top ranked metabolites were not considered to be significantly differential by traditional differential analyses. For example, glutamine, which ranked in 8th in both networks by PROFANCY, was not significantly differential between normal and localized cancer samples or between normal and metastatic cancer samples (Table 3). To explore its association with prostate cancer, we searched lots of literatures. Glutamine is catabolized to glutamate by glutaminase (GLS) and incorporated into citric acid cycle and lipogenesis as an important energy source for proliferation of cancer cells [44], [54]. The glutamine catabolism could be stimulated by oncogenic transcription factor c-MYC to fuel proliferation of cancer cells through up-regulating glutaminase (GLS) [46]. Glutamate has been proved to be a metabolic biomarker of aggressiveness and a potential therapeutic target for prostate cancer [43]. Recently, some newly synthesized glutamine and glutamic acid derivatives were considered as potential novel antitumor agents [42]. Thus, glutamine was a potential prostate cancer related metabolite but not considered to be significantly differential. The reason might be that some cancer-related metabolites, although important in abnormal metabolic process of prostate cancer, only had a subtle change in concentration but could be detected by PROFANCY based on functional similarity. The hierarchical clustering of the profile data revealed that the top 30 ranked metabolites by PROFANCY, although containing quite a part of non-significantly differential metabolites, could effectively classify prostate samples as benign, clinically localized prostate cancer, or metastatic cancer, especially metastatic samples and the other two (Figure 4). The above results suggested that PROFANCY could identify “fine-tuning” disease metabolites which were difficult to be detected by the traditional differential analysis.

## Discussion

In this article, we presented a global method called PROFANCY to prioritize candidate disease-related metabolites based on the assumption that functionally related metabolites tend to associate with the same or similar diseases in the context of metabolic pathway. We first reconstructed a global metabolic network and added functional pathway nodes to fully exploit the modular information. Then we implemented the RWR method on this network. Finally, we could get the rank of the candidate metabolites. The PROFANCY had a good performance on prioritization on 71 diseases and achieved an AUC value up to 0.895. We also applied the PROFANCY on different disease classes and achieved an AUC value over 0.95 in 4 classes. To investigate the robustness of the PROFANCY, we repeated these analyses in another metabolic network reconstructed according to the EHMN database and obtained the similar results. The good performance and robustness were largely attributed to functional pathway nodes. The PROFANCY method also successfully predicted potential novel Alzheimer's disease-related metabolite and prioritized the metabolomics profiles of prostate cancer.

The success of our method could be attributed to the combination of two aspects. Firstly, we took the advantage of the global functional relationships between metabolites. Diseases were usually the consequence of the breakdown of cellular process associated with some functionally related metabolites which were functionally interconnected through metabolic reactions generally grouping into metabolic network [9]. In this study, we used a global distance measure to calculate the similarity between candidate metabolites and known disease metabolites. It was better suited to capture relationships between disease metabolites than the simple algorithms based on direct interactions or shortest paths between disease metabolites [20]. Because current databases of human metabolic network are far from complete. This is clearly problematic for predictions based upon direct interactions with disease metabolites, which would lead to a false-negative/positive prediction. On the contrary, our method based on a global distance measure appeared to be more tolerant of incomplete data. Even when we deleted 20% edges of metabolic network, the AUC value had only a slight decline (Table S3). Our strategy was proved successful in prioritizing known metabolite for 71 diseases with an AUC value up to 0.895. Especially, it had good performance on metabolic-related diseases. Secondly, might be more important, our PROFANCY method sufficiently exploited the functionally modular information of metabolic network. The metabolic network was divided into different metabolic pathways and the metabolites in the same pathway were strongly functionally related [12]. To fully exploit the functional modularity information of metabolic network, we added functional pathway nodes to the metabolic network. The functional pathway nodes would improve the performance by enhancing the connectivity between metabolites related to the same disease, especially for the disease whose metabolites belonged to different pathways. As we mentioned above, two functional pathway nodes enhanced the connectivity of kynurenate and pipecolic acid which were both related to malaria but belonging to different pathways. The results showed that this strategy had effectively improved the performance–three metabolites of malaria were all ranked in top 10% and the AUC for immunological diseases increased from 0.832 to 0.961. The functional pathway nodes also contributed to the robustness of PROFANCY. They could maintain a part of functional relationships between disease metabolites in the incomplete metabolic network. The AUC could achieved to 0.8 even when we removed 70% edges of metabolic network, but this value would declined to 0.65 without functional pathway nodes (Table S3).

We also noticed that there were some limitations of our PROFANCY method. At first, our method depended on the topology of the metabolic network, so the low-quality and incompleteness of reaction information of KEGG or EHMN database might limit its performance. Especially, there were no organ-specific reaction and pathway structure data available currently. Although the PROFANCY could perform well in the incomplete network, the performance could be further improved after more complete and specific reconstructions of metabolic network. Secondly, our result is limited to diseases with known metabolites from the HMDB database and the number of known metabolites might have influence on the performance. Integrating multiple metabolite data sources (for example, from literatures) and availability of well-annotated metabolic pathway may overcome this limitation. The PROFANCY could also be made more flexible not only by using customized seed nodes and candidates but also by fuzzy matching the metabolite names which were supported in our R based or web based tools (http://bioinfo.hrbmu.edu.cn/PROFANCY). It could be expected that PROFANCY would be a beneficial tool for prioritization and prediction of disease metabolites.

## Supporting Information

Figure S1 **The proportion of known disease metabolites in top % rank of all metabolites in (A) KEGG metabolic network and (B) EHMN metabolic network.**
(TIF)

Table S1 **The number of known disease metabolites of 71 diseases in top 10% rank.**
(DOC)

Table S2 **Proportion of known disease metablites in top % rank of all metabolites.**
(DOC)

Table S3 **The AUC value of PROFANCY when deleting edges of metabolic network.**
(DOC)

Table S4 **The AUC value of 16 disease classes by PROFANCY when randomly selecting candidate metabolites.**
(DOC)

Table S5
**Effect of** 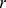 **value.**
(DOC)

Table S6 **Deleted metabolites in the process of reconstructed metabolic network.**
(DOC)
