# Supplementary figures and images for: Prioritizing Candidate Disease Metabolites Based on Global Functional Relationships between Metabolites in the Context of Metabolic Pathways
Source: PLoS One. 2014 Aug 25;9(8):e104934. doi: 10.1371/journal.pone.0104934 (PMC4143229; doi:10.1371/journal.pone.0104934)

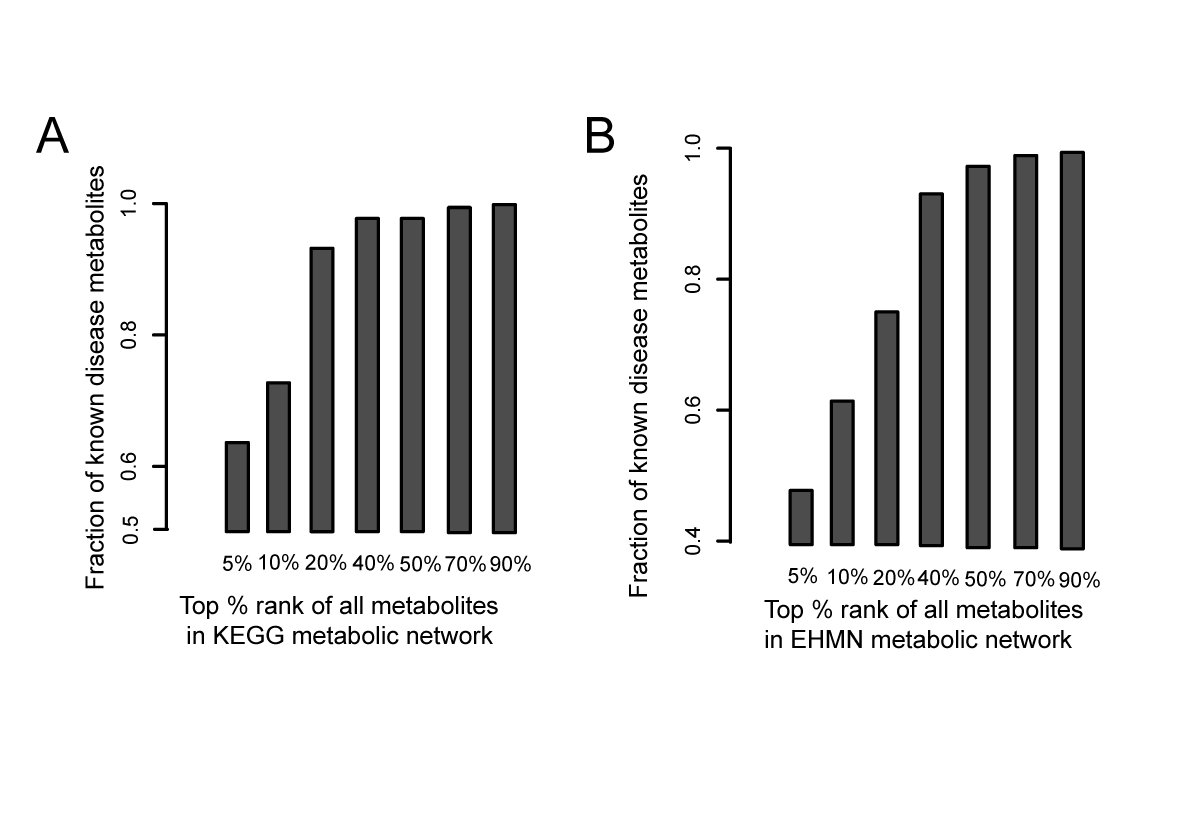

Supplement: Figure S1 — The proportion of known disease metabolites in top % rank of all metabolites in (A) KEGG metabolic network and (B) EHMN metabolic network. (TIF) [file pone.0104934.s001.tif]
