# Supplementary material for: Prioritizing Candidate Disease Metabolites Based on Global Functional Relationships between Metabolites in the Context of Metabolic Pathways
Source: PLoS One. 2014 Aug 25;9(8):e104934. doi: 10.1371/journal.pone.0104934 (PMC4143229; doi:10.1371/journal.pone.0104934)
Supplement: Table S1 — The number of known disease metabolites of 71 diseases in top 10% rank. (DOC) [file pone.0104934.s002.doc]

Table S1 The number of known disease metabolites of 71 diseases in top 10 % rank

| OMIM  ID | OMIM  Name | Disease Class | KDM | EHMN  Without FPN | EHMN  PROFANCY | KEGG Without FPN | KEGG PROFANCY | Union |
| --- | --- | --- | --- | --- | --- | --- | --- | --- |
| 103050 | Adenylosuccinate lyase deficiency | Metabolic | 2 | 1 | 2 | 2 | 2 | 2 |
| 104300 | Alzheimer's disease | Neurological | 44 | 26 | 28 | 32 | 35 | 37 |
| 105800 | Subarachnoid hemorrhage | Cardiovascular | 3 | 2 | 2 | 2 | 2 | 2 |
| 107930 | Aromatic L-amino acid decarboxylase deficiency | Metabolic | 5 | 4 | 4 | 4 | 4 | 4 |
| 109720 | Primary biliary cirrhosis | Metabolic | 3 | 0 | 2 | 2 | 2 | 2 |
| 125853 | Diabetes mellitus type 2 | Endocrine | 7 | 3 | 3 | 4 | 4 | 4 |
| 126200 | Multiple sclerosis | Neurological | 7 | 4 | 5 | 6 | 6 | 6 |
| 139250 | Growth hormone deficiency | Endocrine | 4 | 0 | 0 | 0 | 0 | 0 |
| 140350 | Hawkinsinuria | Metabolic | 2 | 2 | 2 | 2 | 2 | 2 |
| 145500 | Hypertension | Cardiovascular | 6 | 5 | 5 | 5 | 5 | 5 |
| 154800 | Mastocytosis | Immunological | 2 | 2 | 2 | 2 | 2 | 2 |
| 164050 | Purine nucleoside phosphorylase deficiency | Immunological | 4 | 4 | 4 | 4 | 4 | 4 |
| 167320 | Dementia | Muscular | 2 | 1 | 1 | 2 | 2 | 2 |
| 167870 | Panic disorder | Psychiatric | 2 | 2 | 2 | 2 | 2 | 2 |
| 168600 | Parkinson's disease | Neurological | 5 | 2 | 2 | 4 | 4 | 4 |
| 171300 | Pheochromocytoma | Cancer | 3 | 3 | 3 | 3 | 3 | 3 |
| 176807 | Prostate cancer | Cancer | 10 | 2 | 3 | 4 | 5 | 5 |
| 180300 | Rheumatoid arthritis | Connectivetissue | 9 | 6 | 6 | 6 | 6 | 6 |
| 181500 | Schizophrenia | Psychiatric | 22 | 10 | 12 | 17 | 19 | 19 |
| 182601 | Hereditary spastic paraplegia | Neurological | 5 | 2 | 4 | 2 | 4 | 4 |
| 182940 | Spina Bifida | Developmental | 2 | 0 | 0 | 0 | 0 | 0 |
| 184700 | Polycystic ovary syndrome | Metabolic | 2 | 2 | 2 | 2 | 2 | 2 |
| 200100 | Abetalipoproteinemia | Metabolic | 2 | 2 | 2 | 2 | 2 | 2 |
| 201910 | 21-hydroxylase deficiency | Endocrine | 2 | 2 | 2 | 2 | 2 | 2 |
| 207800 | Argininemia | Metabolic | 3 | 3 | 2 | 3 | 3 | 3 |
| 209850 | Autism | Psychiatric | 2 | 0 | 0 | 0 | 0 | 0 |
| 210500 | Biliary atresia | Gastrointestinal | 6 | 6 | 5 | 6 | 6 | 6 |
| 211980 | Lung Cancer | Cancer | 20 | 8 | 9 | 12 | 13 | 13 |
| 214100 | Peroxisomal biogenesis defect | multiple | 2 | 0 | 0 | 0 | 0 | 0 |
| 218700 | Hypothyroidism | Endocrine | 10 | 10 | 10 | 10 | 10 | 10 |
| 219700 | Cystic fibrosis | Respiratory | 3 | 3 | 3 | 3 | 3 | 3 |
| 220100 | Cystinuria | Renal | 4 | 2 | 2 | 2 | 3 | 3 |
| 222690 | Hyperdibasic aminoaciduria I | Metabolic | 3 | 2 | 3 | 2 | 3 | 3 |
| 222700 | Lysinuric protein intolerance | Metabolic | 3 | 2 | 3 | 2 | 3 | 3 |
| 222748 | Dihydropyrimidinase deficiency | Metabolic | 2 | 1 | 2 | 1 | 2 | 2 |
| 229300 | Friedreich's ataxia | Neurological | 5 | 2 | 4 | 2 | 4 | 4 |
| 230400 | Galactosemia type 1 | Metabolic | 3 | 2 | 2 | 2 | 2 | 2 |
| 237300 | Carbamoyl Phosphate Synthetase Deficiency | Metabolic | 4 | 2 | 2 | 2 | 4 | 4 |
| 237400 | Hyper beta-alaninemia | Metabolic | 2 | 2 | 2 | 2 | 2 | 2 |
| 240200 | Addison's Disease | Immunological | 2 | 0 | 0 | 2 | 0 | 2 |
| 242600 | Iminoglycinuria | Metabolic | 3 | 3 | 3 | 3 | 3 | 3 |
| 245130 | 2-ketoadipic acidemia | Metabolic | 2 | 2 | 2 | 2 | 2 | 2 |
| 248310 | Malaria | Immunological | 3 | 1 | 2 | 0 | 3 | 3 |
| 248600 | Maple syrup urine disease | Metabolic | 6 | 6 | 6 | 5 | 6 | 6 |
| 250100 | Metachromatic leukodystrophy | Neurological | 2 | 2 | 2 | 2 | 2 | 2 |
| 261600 | Phenylketonuria | Metabolic | 9 | 8 | 7 | 7 | 7 | 8 |
| 266130 | Glutathione synthetase deficiency | Metabolic | 2 | 1 | 1 | 2 | 2 | 2 |
| 266500 | Refsum's disease | Neurological | 2 | 0 | 0 | 0 | 0 | 0 |
| 266600 | Crohn's disease | Gastrointestinal | 2 | 0 | 2 | 1 | 0 | 2 |
| 270200 | Sjogren-Larsson syndrome | Metabolic | 3 | 3 | 3 | 3 | 3 | 3 |
| 270400 | Smith-Lemli-Opitz syndrome | multiple | 3 | 2 | 2 | 2 | 3 | 3 |
| 271900 | Canavan disease | Metabolic | 15 | 10 | 11 | 12 | 14 | 14 |
| 272300 | Sulfite oxidase deficiency | Metabolic | 2 | 0 | 0 | 1 | 1 | 1 |
| 274270 | Dihydropyrimidine dehydrogenase deficiency | Metabolic | 4 | 4 | 4 | 4 | 4 | 4 |
| 300322 | Lesch-Nyhan syndrome | Metabolic | 5 | 5 | 5 | 4 | 5 | 5 |
| 312750 | Rett syndrome | Neurological | 2 | 1 | 1 | 1 | 1 | 1 |
| 540000 | Stroke | Cardiovascular | 2 | 1 | 1 | 1 | 1 | 1 |
| 601665 | Obesity | Nutritional | 4 | 0 | 0 | 0 | 0 | 0 |
| 602439 | Acute myelogenous leukemia | Hematological | 2 | 0 | 0 | 0 | 0 | 0 |
| 603165 | Eczema | Immunological | 2 | 2 | 2 | 2 | 2 | 2 |
| 604352 | Febrile seizures | Neurological | 2 | 1 | 1 | 1 | 2 | 2 |
| 605899 | D-Glyceric acidemia | Metabolic | 2 | 1 | 2 | 2 | 2 | 2 |
| 606003 | Transaldolase deficiency | Metabolic | 2 | 2 | 2 | 2 | 2 | 2 |
| 606054 | Propionic acidemia | Metabolic | 2 | 2 | 2 | 2 | 2 | 2 |
| 606788 | Anorexia nervosa | Nutritional | 5 | 4 | 4 | 4 | 4 | 4 |
| 606904 | Juvenile myoclonic epilepsy | Neurological | 3 | 3 | 3 | 3 | 3 | 3 |
| 608300 | N-acetylglutamate synthetase deficiency | Metabolic | 2 | 2 | 1 | 2 | 2 | 2 |
| 608516 | Major depressive disorder | Psychiatric | 5 | 2 | 4 | 4 | 4 | 4 |
| 608611 | Ribose-5-phosphate isomerase deficiency | Metabolic | 6 | 4 | 5 | 6 | 5 | 6 |
| 613161 | Beta-ureidopropionase deficiency | Metabolic | 3 | 3 | 3 | 3 | 3 | 3 |
| 613163 | Gaba-transaminase deficiency | Metabolic | 2 | 2 | 2 | 2 | 2 | 2 |
|  | all |  | 338 | 209 | 230 | 245 | 267 | 275 |

KDM= know disease metabolites; FPN=functional pathway nodes
