# Supplementary material for: Prioritizing Candidate Disease Metabolites Based on Global Functional Relationships between Metabolites in the Context of Metabolic Pathways
Source: PLoS One. 2014 Aug 25;9(8):e104934. doi: 10.1371/journal.pone.0104934 (PMC4143229; doi:10.1371/journal.pone.0104934)
Supplement: Table S2 — Proportion of known disease metablites in top % rank of all metabolites. (DOC) [file pone.0104934.s003.doc]

Table S2 Proportion of known disease metablites in top % rank of all metabolites

| Top % rank of all metabolites | Proportion of known disease metablites | | | |
| --- | --- | --- | --- | --- |
|  | EHMN | | KEGG | |
|  | PROFANCY | without FPN | PROFANCY | without FPN |
| 0% | 0 | 0 | 0 | 0 |
| 5% | 0.477273 | 0.386364 | 0.636364 | 0.545455 |
| 10% | 0.613636 | 0.568182 | 0.727273 | 0.681818 |
| 15% | 0.681818 | 0.636364 | 0.886364 | 0.840909 |
| 20% | 0.75 | 0.75 | 0.931818 | 0.909091 |
| 25% | 0.840909 | 0.818182 | 0.931818 | 0.909091 |
| 30% | 0.886364 | 0.818182 | 0.931818 | 0.909091 |
| 35% | 0.931818 | 0.840909 | 0.954545 | 0.909091 |
| 40% | 0.931818 | 0.886364 | 0.977273 | 0.909091 |
| 45% | 0.954545 | 0.886364 | 0.977273 | 0.909091 |
| 50% | 0.977273 | 0.909091 | 0.977273 | 0.954545 |
| 55% | 0.977273 | 0.909091 | 0.977273 | 0.954545 |
| 60% | 0.977273 | 0.909091 | 1 | 1 |
| 65% | 0.977273 | 0.909091 | 1 | 1 |
| 70% | 1 | 0.909091 | 1 | 1 |
| 75% | 1 | 0.931818 | 1 | 1 |
| 80% | 1 | 0.954545 | 1 | 1 |
| 85% | 1 | 1 | 1 | 1 |
| 90% | 1 | 1 | 1 | 1 |
| 95% | 1 | 1 | 1 | 1 |
| 100% | 1 | 1 | 1 | 1 |

FPN = functional pathway nodes
