# Supplementary material for: Prioritizing Candidate Disease Metabolites Based on Global Functional Relationships between Metabolites in the Context of Metabolic Pathways
Source: PLoS One. 2014 Aug 25;9(8):e104934. doi: 10.1371/journal.pone.0104934 (PMC4143229; doi:10.1371/journal.pone.0104934)
Supplement: Table S3 — The AUC value of PROFANCY when deleting edges of metabolic network. (DOC) [file pone.0104934.s004.doc]

Table S3 The AUC value of PROFANCY when deleting edges of metabolic network

| Deleting  % edges | AUC value | | | |
| --- | --- | --- | --- | --- |
|  | EHMN | | KEGG | |
|  | PROFANCY | without FPN | PROFANCY | without FPN |
| 0 | 0.871 | 0.824 | 0.895 | 0.88 |
| 10 | 0.868 | 0.816 | 0.89 | 0.844 |
| 20 | 0.859 | 0.772 | 0.89 | 0.841 |
| 30 | 0.843 | 0.794 | 0.883 | 0.848 |
| 40 | 0.827 | 0.782 | 0.874 | 0.8 |
| 50 | 0.834 | 0.726 | 0.882 | 0.778 |
| 60 | 0.816 | 0.713 | 0.849 | 0.704 |
| 70 | 0.788 | 0.715 | 0.804 | 0.653 |
| 80 | 0.677 | 0.66 | 0.72 | 0.619 |
| 90 | 0.646 | 0.583 | 0.644 | 0.657 |

FPN = functional pathway nodes
