# Supplementary material for: Prioritizing Candidate Disease Metabolites Based on Global Functional Relationships between Metabolites in the Context of Metabolic Pathways
Source: PLoS One. 2014 Aug 25;9(8):e104934. doi: 10.1371/journal.pone.0104934 (PMC4143229; doi:10.1371/journal.pone.0104934)
Supplement: Table S4 — The AUC value of 16 disease classes by PROFANCY when randomly selecting candidate metabolites. (DOC) [file pone.0104934.s005.doc]

Table S4 The AUC value of 16 disease classes by PROFANCY when randomly selecting candidate metabolites

| Disease class | AUC value | |
| --- | --- | --- |
|  | KEGG | EHMN |
| Metabolic | 0.957 | 0.936 |
| Neurological | 0.901 | 0.861 |
| Cardiovascular | 0.844 | 0.842 |
| Endocrine | 0.866 | 0.831 |
| Immunological | 0.96 | 0.91 |
| Muscular | 0.955 | 0.801 |
| Psychiatric | 0.872 | 0.85 |
| Cancer | 0.885 | 0.787 |
| Connective tissue | 0.839 | 0.797 |
| Developmental | 0.561 | 0.578 |
| Gastrointestinal | 0.823 | 0.942 |
| Multiple | 0.633 | 0.767 |
| Respiratory | 1 | 0.982 |
| Renal | 0.931 | 0.88 |
| Nutritional | 0.647 | 0.671 |
| Hematological | 0.444 | 0.614 |
| All | 0.895 | 0.87 |
