# Supplementary material for: Prioritizing Candidate Disease Metabolites Based on Global Functional Relationships between Metabolites in the Context of Metabolic Pathways
Source: PLoS One. 2014 Aug 25;9(8):e104934. doi: 10.1371/journal.pone.0104934 (PMC4143229; doi:10.1371/journal.pone.0104934)
Supplement: Table S5 — Effect of value. (DOC) [file pone.0104934.s006.doc]

Table S5 Effect of $r$ value

| $r$value | AUC value | |
| --- | --- | --- |
|  | EHMN | KEGG |
| 0.1 | 0.882 | 0.908 |
| 0.3 | 0.879 | 0.901 |
| 0.5 | 0.875 | 0.897 |
| 0.7 | 0.871 | 0.895 |
| 0.9 | 0.863 | 0.89 |
