# Supplementary material for: Prioritizing Candidate Disease Metabolites Based on Global Functional Relationships between Metabolites in the Context of Metabolic Pathways
Source: PLoS One. 2014 Aug 25;9(8):e104934. doi: 10.1371/journal.pone.0104934 (PMC4143229; doi:10.1371/journal.pone.0104934)
Supplement: Table S6 — Deleted metabolites in the process of reconstructed metabolic network. (DOC) [file pone.0104934.s007.doc]

Table S6 Deleted metabolites in the process of reconstructed metabolic network

| KEGG ID | HMDB ID | Name |
| --- | --- | --- |
| C00001 | HMDB02111 | Water |
| C00002 | HMDB00538 | Adenosine triphosphate |
| C00003 | HMDB00902 | NAD |
| C00004 | HMDB01487 | NADH |
| C00005 | HMDB00221 | NADPH |
| C00006 | HMDB00217 | NADP |
| C00007 | HMDB01377 | Oxygen |
| C00008 | HMDB01341 | ADP |
| C00009 | HMDB01429 | Phosphate |
| C00010 | HMDB01423 | Coenzyme A |
| C00011 | HMDB01967 | Carbon dioxide |
| C00012 |  | Peptide |
| C00013 | HMDB00250;HMDB02142 | Pyrophosphate;Phosphoric acid |
| C00014 | HMDB00051 | Ammonia |
| C00016 | HMDB01248 | FAD |
| C00017 |  | Protein |
| C00023 | HMDB00692 | Iron |
| C00026 | HMDB00208 | Oxoglutaric acid |
| C00027 | HMDB03125 | Hydrogen peroxide |
| C00028 |  | Acceptor;Hydrogen-acceptor |
| C00030 |  | Reduced acceptor;AH2 |
| C00034 | HMDB01333 | Manganese |
| C00038 | HMDB01303 | Zinc |
| C00045 |  | Amino acid;Amino acids |
| C00050 |  | Metal |
| C00059 | HMDB01448 | Sulfate |
| C00060 |  | Carboxylate |
| C00069 |  | Alcohol |
| C00070 | HMDB00657 | Copper |
| C00076 | HMDB00464 | Calcium |
| C00080 |  | H+;Hydron |
| C00087 | HMDB00598 | Sulfide |
| C00088 | HMDB02786 | Nitrite |
| C00089 | HMDB00258 | Sucrose |
| C00098 |  | Oligopeptide |
| C01328 | HMDB01039 | Hydroxide |
| C14819 | HMDB12943 | Fe3+ |
